# Functional Connectivity of Language-Related Cerebellar Regions Is Reduced in Schizophrenia Patients

**DOI:** 10.3390/biomedicines12030480

**Published:** 2024-02-21

**Authors:** Marco Marino, Margherita Biondi, Dante Mantini, Chiara Spironelli

**Affiliations:** 1 Department of General Psychology, University of Padova, 35131 Padova, Italy; marco.marino@unipd.it; 2 Movement Control and Neuroplasticity Research Group, KU Leuven, 3001 Leuven, Belgium; dante.mantini@kuleuven.be; 3 Padova Neuroscience Center, University of Padova, 35131 Padova, Italy; margherita.biondi.1@phd.unipd.it

**Keywords:** brain networks, cerebellum, cerebellar parcellation, schizophrenia, seed-based connectivity, cognitive symptoms

## Abstract

Schizophrenia (SZ) is a widespread psychiatric disorder that is traditionally characterized by positive and negative symptoms. However, recent focus has shifted to cognitive deficits as a crucial aspect. The cerebellum, conventionally tied to motor coordination, is now recognized as pivotal in the pathophysiology of SZ cognitive impairments. Proposed disruptions in the cortico-cerebellar-thalamic-cortico circuit contribute to these deficits. Despite evidence of cerebellar abnormalities, within-cerebellum functional connectivity is often overlooked. This study explores spontaneous functional interactions within the cerebellum and their link to cognitive deficits in SZ. Using a multi-domain task battery (MDTB) parcellation, fMRI data from SZ patients and healthy controls were analyzed. Significant differences in cerebellar connectivity emerged, particularly in regions related to attention, language, and memory processing. Correlations between connectivity values and SZ symptomatology were identified. A post hoc analysis, considering the patients’ hallucination vulnerability, revealed distinct connectivity patterns. Non-hallucinating and low-hallucinating SZ patients exhibited higher cerebellar connectivity than high-hallucinating patients, especially in language and motor control regions. These findings suggest a gradient of cerebellar connectivity alterations corresponding to hallucination vulnerability in SZ patients. This study offers novel insights into cerebellar impairments in SZ, highlighting the role of within-cerebellum connectivity in cognitive deficits. The observed connectivity patterns in language-related regions contribute to understanding language development and auditory verbal hallucinations in SZ.

## 1. Introduction

Schizophrenia (SZ) is a severe and complex psychiatric disorder affecting approximately 20 million individuals worldwide [1]. This condition is associated with heterogeneous and extensive impairments that strongly affect quality of life, general health, functioning, and subjective well-being [2]. Being considered for many years the archetype of psychotic illness [3], SZ diagnostic characterization has been primarily traced to the presence of positive (e.g., hallucinations, delusions) and/or negative symptoms (e.g., affective flattening, social withdrawal), which consequently became the main target for research and treatment [4,5]. However, the presence of a third core symptoms category in SZ has been more recently established and has gained clinical and research relevance: cognitive deficits [5]. Their key role in SZ has been identified following the increasing evidence in favor of their independent etiology. Cognitive deficits do not merely result from positive or negative symptoms, and their emergence can only be partially ascribed as side effects of treatment [6]. Notably, epidemiological data show that most SZ patients suffer from a prominent neurocognitive impairment [7] and that, compared to healthy people, their average level of overall cognitive functioning is approximately two standard deviations lower [8], especially in the domains of attention, executive functioning, processing speed, social cognition, and working, verbal, and visual memory [9].

Among the putative brain regions that seem to play a key role in the pathophysiology of cognitive deficits in SZ [10], the cerebellum has gained particular interest mainly due to the reconceptualization of its involvement in cognition [11]. Indeed, the last decades of research have shown that the cerebellum, in addition to its well-known role in motor coordination [12], is involved in cognitive performance [13]. Therefore, the disruption of its functioning, occurring in various neurological [14] and psychiatric conditions, foremost SZ [15], might lead to cognitive deficits. Nancy Andreasen’s work has been crucial to highlight the cerebellar dysfunction characterizing SZ: starting from positron emission tomography (PET) evidence showing that SZ patients had significantly reduced cerebral blood flow in cerebellar, thalamic, and prefrontal areas during cognitive tasks compared to healthy individuals [16], Andreasen and colleagues hypothesized that the well anatomically described cortico-cerebellar-thalamic-cortico circuit (CCTCC) [17], when altered, could be the neurobiological source of cognitive deficits in SZ patients [18]. This founds the basis of the “cognitive dysmetria theory”, which states that, in a physiological condition, cerebellum, thalamus, and prefrontal cortex fluidly collaborate to orchestrate cognitive abilities—similarly to how motor activities are sustained—and when this loop is disrupted, cognitive dysmetria might arise, resulting in difficulty coordinating different cognitive functions and leading to a multitude of deficits in individuals with this disorder [19]. In a subsequent publication focusing on the role of the cerebellum in SZ, Andreasen suggested the importance of cerebellum malfunctioning as a possible etiopathogenetic explanation of cognitive symptoms in this disorder. Still, their investigation focused on the cerebellum in relation to its long-range connections and involvement with cortical regions [20] rather than local communication within the cerebellum itself. In particular, the opposite contributions—inhibitory and excitatory—of the Purkinje and the granule cells, respectively, appeared critical to modulate and/or coordinate cortical activity. Furthermore, the structural and functional connections of the Purkinje cells of cerebellum are significantly involved in the processing of specific input, e.g., detection of changes in spatial location or in auditory stimulation [20]. With their inhibitory effect, these fibers mediate which information should be returned to the cortex. A deficiency of Purkinje cells’ inhibitory effects might contribute to misinterpretations of sensory stimuli, leading to psychotic symptoms [18,19,21].

Evidence of abnormalities in the CCTCC across SZ patients with cognitive impairments has been reported over the past decades [22,23], highlighting cerebellar structural anomalies, e.g., references [24,25], as well as structural and functional cortico-cerebellar connectivity alterations, e.g., references [26,27,28], in these patients. Nevertheless, while findings accounting for cerebellar abnormalities are consistent from some perspectives, such as neurological soft signs and posture, they are highly heterogeneous from others, including cognition [23]. In addition, the functional connectivity within the cerebellum itself has often been neglected. Several studies carried out on healthy participants have demonstrated the involvement of the cerebellum in a wide range of cognitive domains, including—among others—motor functions, attention, language, and memory, which are known to be impaired in SZ, e.g., references [29,30,31]. With a multi-domain task battery (MDTB) of 26 tasks and 47 conditions, King et al. (2019) [32] trained and tested 24 healthy individuals to define the functional specialization of each cerebellar region. Their new comprehensive parcellation of the cerebellar cortex, based on motor, cognitive, and emotional/affective functions, offers an important tool to investigate cerebellar interactions and identify their relationship with cognition. Thus, by exploring the connectivity among cerebellar subregions and its association with patients’ cognitive profiles, this new atlas may contribute to the definition of the pathophysiological role of the cerebellum in SZ.

In this study, we aimed to investigate spontaneous functional interactions in the cerebellum and to identify their relationship with cognitive deficits in SZ. To this end, we applied the MDTB parcellation of the cerebellum [32] to functional magnetic resonance imaging (fMRI) data collected from SZ patients during the resting state. We examined whether functional connectivity alterations between specific cerebellar regions were associated with this disorder and identified which and to what extent cerebellar functional alterations may underlie SZ cognitive symptoms.

## 2. Materials and Methods

### 2.1. Participants

The fMRI data are stored in SchizConnect (http://schizconnect.org, accessed on 10 February 2021), a public and virtual database for research in neuroimaging. The platform contains data from various databases, including the Center for Biomedical Research Excellence (COBRE), MCIC (available through the COINS database), Functional Biomedical Informatics Research Network (FBIRN), XNAT Central, and NUNDA and Research Electronic Data Capture (REDCap) frameworks from Northwestern University. Access to such heterogeneous data allows us to conduct large-scale studies, carrying out multi-site, multidimensional, and multimodal analyses. Our data sample of SZ patients and healthy controls (HCs) met the following requirements: (1) availability of resting-state fMRI images data; (2) availability of T1 structural MRI (sMRI) data; and (3) availability of the complete scores of the positive and negative syndrome scale (PANSS) [33] for the SZ sample only. The dataset used in the present study was shared in the domain of the COBRE project, which collects data from psychiatric patients attending the UNM Psychiatric Center, the Raymond G. Murphy Veterans Affairs Medical Center, and other clinics in the Albuquerque metropolitan area (USA). The COBRE project criteria for SZ patient selection were: (1) a SZ diagnosis, confirmed by two different psychiatrists using the DSM-IV Structured Clinical Interview for Axis I disorders (SCID) [34]; (2) clinical stability, assessed both 3 months before the experimental session and during MRI sessions; and (3) an age between 18 and 65 years old. HCs were also recruited in Albuquerque. To be sure to enroll only healthy adults, strict exclusion criteria based on the SCID-Non Patient administration within the COBRE project were applied, i.e., (1) diagnosis of an ongoing or past Axis I psychiatric disorder; (2) head trauma (with loss of consciousness greater than 5 min); (3) recent history of substance abuse, addiction, or antidepressant use in the past 5 months; (4) having a first-degree relative with a psychotic disorder.

All the participants involved in the COBRE project provided written informed consent. The Collaborative Informatics and Neuroimaging Suite Data Exchange (COINS; http://coins.mrn.org/dx, accessed on 10 February 2021) made data downloading possible. COBRE-funded data collection took place at the Mind Research Network, subject to the licensing procedure 5P20RR021938/P20GM103472 from the National Institutes of Health (NIH) to Dr. Vince Calhoun. All the data were anonymized before access to protect the privacy of the participants. Structural and resting-state images were further visually inspected to check for sample suitability. The final dataset consisted of 74 SZ patients and 74 HC, similar for age (mean ± standard deviation: 37.43 ± 14.02 and 37.88 ± 12.69 years, respectively; *t*_146_ = 0.20, n.s.) and gender distribution (59 SZ men and 15 SZ women and 54 HC men and 20 HC women; *χ*^2^ = 0.93, n.s.).

### 2.2. MRI Data Acquisition

MRI data were acquired using a 3T Siemens MR scanner (Trio, Siemens Healthcare, Erlangen, Germany). In order to obtain image slices that were axial, oblique, and parallel to the antero-posterior commissure (AC-PC) line, the acquisition protocol allowed for sagittal-gradient echo-scout images through the midline. In particular, oblique slices were used to minimize the orbitofrontal susceptibility artifact. fMRI data were collected using a single-shot gradient echo-planar pulse sequence with lipid suppression using the following parameters: TR = 2000 ms, TE = 29 ms, flip angle = 75°, FOV = 240 mm, matrix size = 64 × 64, 33 slices, voxel size = 3.75 × 3.75 × 4.55 mm^3^, number of volumes = 150. In addition, sMRI data (high-resolution T1-weighted) were collected using a multi-echo MP-RAGE sequence (5 echoes) using the following parameters: TE = 1.64, 3.5, 5.36, 7.22, 9.08 ms, TR = 2.53 s, TI = 1.2 s, flip angle = 7°, NEX = 1, slice thickness = 1 mm, FOV = 256 mm, resolution = 256 × 256. The first image of each acquisition was removed to account for T1 equilibrium effects. The scanning parameters are described in detail at the following link: http://schizconnect.org/uploads/data_instruction/pdffile/2/COBRE_Scan_Information.pdf (last accessed on 10 February 2021).

### 2.3. MRI Data Preprocessing

**Figure 1:**
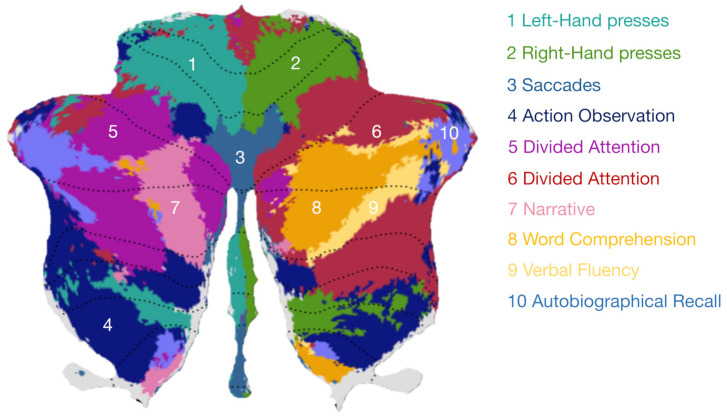
Cognitive descriptors for the MDTB parcellation proposed by King et al. (2019) [32]. The most prominent features that best characterize each region are listed and include: (1) left-hand presses (light green), (2) right-hand presses (dark green), (3) saccades (blue marine), (4) action observation (dark blue), (5) divided attention (purple), (6) divided attention (red), (7) narrative (pink), (8) word comprehension (orange), (9) verbal fluency (yellow), and (10) autobiographical recall (light blue). The figure was generated using the image viewer from the Diedrichsen Lab (https://www.diedrichsenlab.org/imaging/AtlasViewer/, accessed on 10 September 2023).

The fMRI data were preprocessed using an automated pipeline developed using the Statistical Parametric Mapping 12 (SPM12) software (https://www.fil.ion.ucl.ac.uk/spm/software/spm12/, accessed on 10 September 2023) in the MATLAB^®^ (MathWorks Inc., Natick, MA, USA) environment. This pipeline included motion correction, spatial alignment to the structural image, band-pass filtering (0.01–0.1 Hz), white matter, cerebrospinal fluid and global signals regression, and spatial smoothing at a 6 mm full-width half maximum (FWHM) [35]. Regions of interest (ROIs) were selected based on the cerebellar parcellations proposed by King et al. (2019) [32] (Figure 1). The multi-domain task battery (MDTB) parcellation allowed us to define reliable functional boundaries in the cerebellum. In our study, we used a ten-region parcellation which has been shown to provide a useful level of resolution for full functional characterization [32]. In their study, King and colleagues identified cognitive descriptors for the 10 functional regions in the MDTB parcellation (Figure 1), which are primarily representative of the following cognitive descriptors: (1) left-hand presses—1LH, (2) right-hand presses—2RH, (3) saccades—3S, (4) action observation—4AO, (5) divided attention—5DA, (6) divided attention—6DA, (7) narrative—7N, (8) word comprehension—8WC, (9) verbal fluency—9VF, and (10) autobiographical recall—10AR.

### 2.4. fMRI Connectivity Analysis

We examined the functional connectivity between different pairs of the 10 ROIs. At the individual level, the time-series across all voxels within each of the 10 ROIs were averaged, and the Pearson correlation coefficients among all the ROIs were computed [36]. This analysis yielded a 10 × 10 connectivity matrix. Each correlation coefficient r was converted to z-values using Fisher’s r-to-z transformation. The fMRI connectivity analysis was first performed by considering both the HC and SZ groups to identify differences in functional connectivity between healthy and pathological populations. To assess differences in connectivity between the two groups, a two-tailed paired *t*-test was performed on the connectivity values for each ROI. The false discovery rate (FDR) method [37] was used to account for multiple comparisons across ROIs, and the significance level was set to *q* < 0.05.

### 2.5. Correlation between fMRI Connectivity Values and PANSS Scores

To probe the existence of a relationship between the affective/cognitive symptoms of SZ and the connectivity in the patients’ cerebella, we computed the Spearman’s correlation coefficients between the PANSS scores and each ROI connectivity value corresponding to each MDTB region. The significance level of the correlations was set to *p* < 0.05 (uncorrected) and to *q* < 0.05 (FDR corrected).

## 3. Results

### 3.1. Cerebellar MDTB-Based Connectivity Analysis

**Figure 2:**
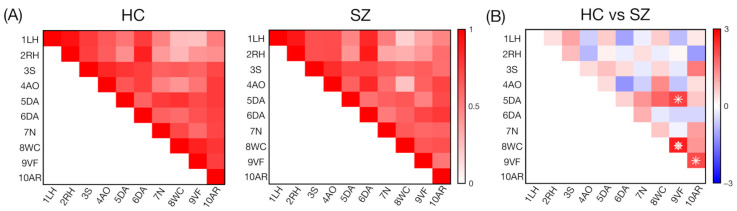
Functional connectivity values (and their differences) between all possible pairs of ROIs for the HC and SZ groups. (**A**) The matrices represent the average values for the HC and SZ groups. In each panel, the values on the diagonal correspond to the average connectivity within that specific ROI, whereas the values on the upper triangular matrix correspond to the average connectivity between pairs of ROIs, where the ten ROIs from the MDTB parcellation used are as follows: (1) left-hand presses—1LH, (2) right-hand presses—2RH, (3) saccades—3S, (4) action observation—4AO, (5) divided attention—5DA, (6) divided attention—6DA, (7) narrative—7N, (8) word comprehension—8WC, (9) verbal fluency—9VF, and (10) autobiographical recall—10AR. (**B**) For each ROI pair, significant differences between the HC and SZ groups at *p* < 0.05 are marked with an asterisk, and those at *q* < 0.05 are marked with a diamond.

We found significant differences in the connectivity values between the HC and SZ groups for several ROIs (Figure 2A), spanning cerebellar regions linked to attention (5DA), language (8WC and 9VF), and memory processing (10AR). Higher connectivity values were reported for the HC group compared to the SZ group for the 5DA-9VF, 8WC-9VF (FDR corrected), and 9VF-10AR pairs (Figure 2B).

When exploring the involvement of these specific connections with SZ symptoms, we identified significant negative relationships between the connectivity values of attention- and language-related regions and many items of the General Psychopathology Scale, including the GP2 (anxiety), GP3 (guilt feelings), GP4 (tension), and GP6 (depression) items, as well as a significant positive relationship between 10AR and the N1 item of the PANSS. Correlation plots for all significant correlations are reported in Figure S1 (Supplementary Materials).

### 3.2. Post Hoc Cerebellar MDTB-Based Connectivity Analysis of Patient Subgroups

**Figure 3:**
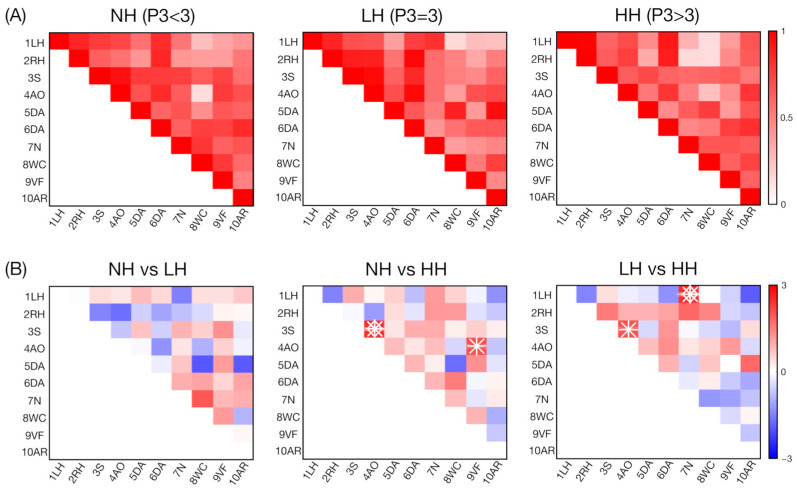
Functional connectivity values (and their differences) between all possible pairs of ROIs for the SZ subgroups. (**A**) The matrices represent the average values for the SZ subgroups, including non-hallucinating (NH), low-hallucinating (LH), and high-hallucinating (HH) patients. In each panel, the values on the diagonal correspond to the average connectivity within that specific ROI, whereas the values on the upper triangular matrix correspond to the average connectivity between pairs of ROIs, where the ten ROIs from the MDTB parcellation used are as follows: (1) left-hand presses—1LH, (2) right-hand presses—2RH, (3) saccades—3S, (4) action observation—4AO, (5) divided attention—5DA, (6) divided attention—6DA, (7) narrative—7N, (8) word comprehension—8WC, (9) verbal fluency—9VF, and (10) autobiographical recall—10AR. (**B**) For each ROI pair, significant differences between SZ subgroups at *p* < 0.05 are marked with an asterisk and those at *q* < 0.05 are marked with a diamond.

Considering the significant, FDR-corrected, decreased connectivity values reported for the SZ group compared to the HC group for the word comprehension (8WC) and verbal fluency (9VF) ROIs, we decided to carry out an additional analysis focused on patient subgroups. Indeed, according to Crow’s hypothesis [38,39], reduced left hemisphere dominance for language represents a risk factor for SZ, and we found evidence of a direct association between altered language lateralization and verbal hallucinations in the SZ patients [40,41,42]. Therefore, we hypothesized that decreased connectivity in the cerebellar ROIs engaged in language-related processing, such as 8WC and 9VF, could be reasonably associated with the hallucinatory phenomena of SZ patients. With this in mind, we divided the patient group into three subgroups according to their vulnerability to hallucinate based on the P3 (hallucinatory behavior) item of the PANSS. In particular, the patients in the three subgroups were mutually exclusive, such that if a patient obtained a P3 PANSS score < 3, =3, >3, the patient was assigned to the non-hallucinating (NH, *n* = 28), low-hallucinating (LH, *n* = 17), and high-hallucinating (HH, *n* = 29) subgroups, respectively. To assess differences in connectivity between pairs of subgroups, a two-tailed paired *t*-test was performed for the connectivity values for each ROI so that three contrasts (NH vs. LH, NH vs. HH, and LH vs. HH) were computed. FDR (*q* < 0.05) was used to account for multiple comparisons. A correlation analysis with affective and cognitive symptoms (assessed with the PANSS scale) was also separately performed for each subgroup to identify specific relationships depending on the patients’ vulnerability to hallucinate. The results of this post hoc patient grouping revealed significant differences in the connectivity values between the different SZ subgroups for specific ROIs (Figure 3A) including (associative) motor regions (1LH, 3S, and 4AO) and language-related regions (7N, and 9VF). Higher connectivity values were reported for the NH group compared to the HH group for the 3S-4AO (FDR corrected) and 4AO-9VF pairs (Figure 3B). Higher connectivity values were reported for the LH group compared to the HH group for the 3S-4AO (similar to the NH and HH comparison, but uncorrected) and 1LH-7N pairs (FDR corrected) (Figure 3B). No significant differences were reported between the NH and LH groups.

Considering the associations of these specific connections with PANSS, we found relatively strong relationships, ranging from −0.60 to 0.57 for positive and negative correlations, respectively. In particular, we reported positive associations between 3S-4AO and the GP1 (somatic concern), GP9 (unusual thought content), and P5 (grandiosity) items and negative associations between 1LH-7N and the GP2 (anxiety), GP5 (mannerisms and posturing), and GP14 (poor impulse control) items, depending on the subgroups. Correlation plots for all the significant correlations are reported in Figure S2 (Supplementary Materials).

## 4. Discussion

A recent study revealed that—beyond its core motor functioning involvement—the cerebellum is engaged in a range of cognitive functions, including attentional processing, language, executive functions, and working memory [32]. Previous studies have shown a link between altered cortico-cerebellar connectivity in fluid intelligence [43] and in the processing of error information for performance optimization [44] in healthy individuals as well as in SZ patients. Still, to the best of our knowledge, within-cerebellum connectivity has not been explored in SZ. In the present study, we addressed this research gap by relying on the MDTB parcellation to gain new insights into cerebellar impairments in SZ and their relation to compromised cognitive functions (Figure 2A). We found that, compared with the HC group, the SZ patients exhibited decreased cerebellar connectivity in two regions associated with distinct linguistic functions, i.e., word comprehension (8WC) and verbal fluency (9VF) (Figure 2B). Both of these ROIs are located in the lobulus semilunaris inferior of the right cerebellar hemisphere, one next to the other (Figure 1).

Reduced left hemisphere dominance for language processing represents a risk factor for SZ [38,39], and altered language lateralization has been widely reported in both SZ, e.g., references [45,46,47,48,49,50], as well as other functional psychosis, such as bipolar disorder, e.g., references [51,52]. In line with these findings, decreased connectivity in the cerebellar ROIs engaged in language-related processing, such as 8WC and 9VF, could represent another result supporting the framework of Crow’s model. In addition, recent studies provided evidence of a direct association between bilaterally distributed language lateralization and the severity of verbal hallucinations in SZ patients [40,41,42]. For this reason, we posit that the patients’ decreased cerebellar connectivity in word comprehension and verbal fluency ROIs could also be reasonably associated with the hallucinatory phenomena of SZ patients. We therefore carried out a post hoc analysis, considering patient subgroups according to their vulnerability to hallucination, by considering the P3 (hallucinatory behavior) item of the PANSS (Figure 3A). Interestingly, no connectivity differences were found when considering the non-hallucinating (NH) and low-hallucinating (LH) patients, whereas both these subgroups revealed significantly higher cerebellar connectivity values when compared with high-hallucinating (HH) SZ patients (Figure 3B). We identified a connectivity gradient between the 3S and 4AO regions, as the HH subgroup showed decreased connectivity compared with both the LH (uncorrected) and NH (FDR-corrected) subgroups. These two regions are located in the declive (vermis) and the left lobules biventer (H VIII A, H VIII B) and have been associated, beyond saccadic movements and action observation, with visual letter recognition and visual working memory functions (3S ROI) and motor planning and divided attention tasks (4AO ROI) [32], respectively.

Altered saccadic eye movements have been consistently found in SZ patients [53]; therefore, some authors have considered this defect as a possible disease marker [54]. Interestingly, Subramaniam et al. (2018) [55] investigated the association between saccadic performance and clinical symptoms in drug-free SZ patients. In particular, compared with healthy controls, SZ patients showed smaller (in amplitude) antisaccades and they made more errors, resulting in more severe hallucinations. Therefore, by describing the relationship between antisaccade errors and hallucination severity, a potential link was identified between hallucinations and deficits in inhibitory control, which, in light of our findings, might lie in altered cerebellar functioning. Interestingly, we found significant differences for cerebellar connections supporting narrative comprehension (region 7), language functions (regions 8 and 9), and autobiographical recall (region 10), which are linked to regions belonging to the default mode network (DMN) such as the left angular gyrus (lAG), which notably is involved in inner speech monitoring [56,57], language [58], and memory and/or self-referential [59] processing. Importantly, alterations in lAG functioning have been reported in SZ and associated with the vulnerability to hallucinate [56,60].

In our findings, we reported further differences between the HH groups compared to the ones presenting limited or no vulnerability to hallucination. We found that the LH SZ patients exhibited higher cerebellar connectivity between the 1LH and 7N ROIs compared with the HH patients. These regions, both located in the left cerebellar hemisphere, are involved in motor planning (1LH) and emotion and language processing (7N). Furthermore, the NH SZ patients had higher cerebellar connectivity between the 4AO and 9VF ROIs compared with the HH patients. For this comparison, this also suggests that the SZ patients characterized by a higher vulnerability to hallucinate presented an altered functional interaction between regions involved in motor control and language processing.

An exploratory direct comparison between the HC group and the SZ subgroups was also performed. This analysis demonstrated that the differences between the HC and the SZ groups were partially ascribed to the HH group (Figure S3), as the 5DA-9VF and 8WC-9VF pairs persisted in the subgroup comparison. At the same time, a strong difference also persisted between the HC and the LH groups for the 8WC-9VF pair. This eventually confirmed the possible key role of these cerebellar regions in the emergence of auditory verbal hallucinations in SZ. Also, the difference in connectivity between the 3S-4AO pair in the HC and HH group comparison (which was also reported for the NH vs. HH and LH vs. HH comparisons) suggests that altered connectivity between these regions might underlie hallucinatory phenomena. While our findings highlight the possible role of the cerebellum in language processing, we cannot state that this evidence could demonstrate a clear correspondence with specific symptoms in schizophrenia, e.g., auditory verbal hallucinations. To this end, further investigations involving dedicated language tasks could clarify the association between cerebellar functioning and clinical symptoms. By investigating the role of cerebellum in language, future studies could provide crucial information to understand the development and/or to assess the eventual deficits of language-related skills, which applies not only to psychiatric but also to developmental disorders such as dyslexia [61]. Indeed, dyslexia patients, similarly to SZ, display dysfunctional language lateralization [62] which might arise from alterations in the interactions within the cerebellum and between the cerebellum and the cortex by involving pathways linked to both motor control and cognitive processing [63,64].

## 5. Conclusions

In conclusion, in this study, we provided the first evidence that functional connectivity of language-related cerebellar regions is reduced in SZ patients. The framework of investigation could be further expanded in future studies by considering specific cerebellar–cortical connections to gain further insights to characterize the relationship between alterations in cerebellar functioning, language processing, and auditory verbal hallucinations in SZ.

## Data Availability

The raw data supporting the conclusions of this article were downloaded from Schiz-Connect (http://schizconnect.org, accessed on 10 February 2021), a public and virtual database for research purposes.

## Supplementary Materials

The following supporting information can be downloaded at: https://www.mdpi.com/article/10.3390/biomedicines12030480/s1, Figure S1: Correlation plots for significant relationships between fMRI connectivity values and PANSS scores for SZ patients. The analysis was conducted for the ROIs that presented significant differences between the HC and SZ groups. Spearman’s correlation coefficients were calculated (*p* < 0.05, uncorrected). Significant negative relationships between connectivity values of attention- and language-related regions and many items of the General Psychopathology Scale were found, including the GP2 (Anxiety), GP3 (Guilt feelings), GP4 (Tension), and GP6 (Depression) items. A significant positive relationship between 10AR and the N1 (Blunted affect) item of the PANSS was also found. Figure S2: Correlation plots for significant relationships between fMRI connectivity values and PANSS scores for SZ patients, separately considered according to their subgroups. The analysis was conducted for the ROIs that presented significant differences between the SZ subgroups, including Low-Hallucinating (LH) and High-Hallucinating (HH) SZ patients. Spearman’s correlation coefficients were calculated (*p* < 0.05, uncorrected). Positive associations between 3S-4AO and the GP1 (Somatic concern), GP9 (Unusual thought content), and P5 (Grandiosity) items, and negative associations between 1LH-7N and the GP2 (Anxiety), GP5 (Mannerisms and posturing), and GP14 (Poor impulse control) items, depending on the subgroups, were found. Figure S3: Functional connectivity differences between all possible pairs of ROIs for the HC group and the SZ subgroups, i.e., Non-Hallucinating (NH), Low-Hallucinating (LH) and High-Hallucinating (HH). The ten ROIs from the MDTB parcellation were used, and included (1) left-hand presses—1LH, (2) right-hand presses—2RH, (3) saccades—3S, (4) action observation—4AO, (5) divided attention—5DA, (6) divided attention—6DA, (7) narrative—7N, (8) word comprehension—8WC, (9) verbal fluency—9VF, and (10) autobiographical recall—10AR. For each ROI pair, significant differences between HC group and SZ subgroups at *p* < 0.05 are marked with an asterisk, those at *q* < 0.05 with a diamond.
