# Supplementary material for: Functional Connectivity of Language-Related Cerebellar Regions Is Reduced in Schizophrenia Patients
Source: Biomedicines. 2024 Feb 21;12(3):480. doi: 10.3390/biomedicines12030480 (PMC10968155; doi:10.3390/biomedicines12030480)
Supplement: Supplementary file 1 [file biomedicines-12-00480-s001.zip › biomedicines-2874347-supplementary.pdf]

Supplementary materials for

Functional connectivity of language-related cerebellar regions is reduced in schizophrenia patients

Marco Marino, Margherita Biondi, Dante Mantini, and Chiara Spironelli

Correlation between cerebellar MDTB-based connectivity analysis and PANSS scores for SZ patients

To probe the existence of a relationship between SZ affective/cognitive symptoms and connectivity in patients' cerebellum, we computed the Spearman's correlation coefficients between the PANSS scores and each ROI connectivity values corresponding to each MDTB region. Correlation plots in Fig. S1 depict the significant relationships obtained with $p < 0.05$ (uncorrected).

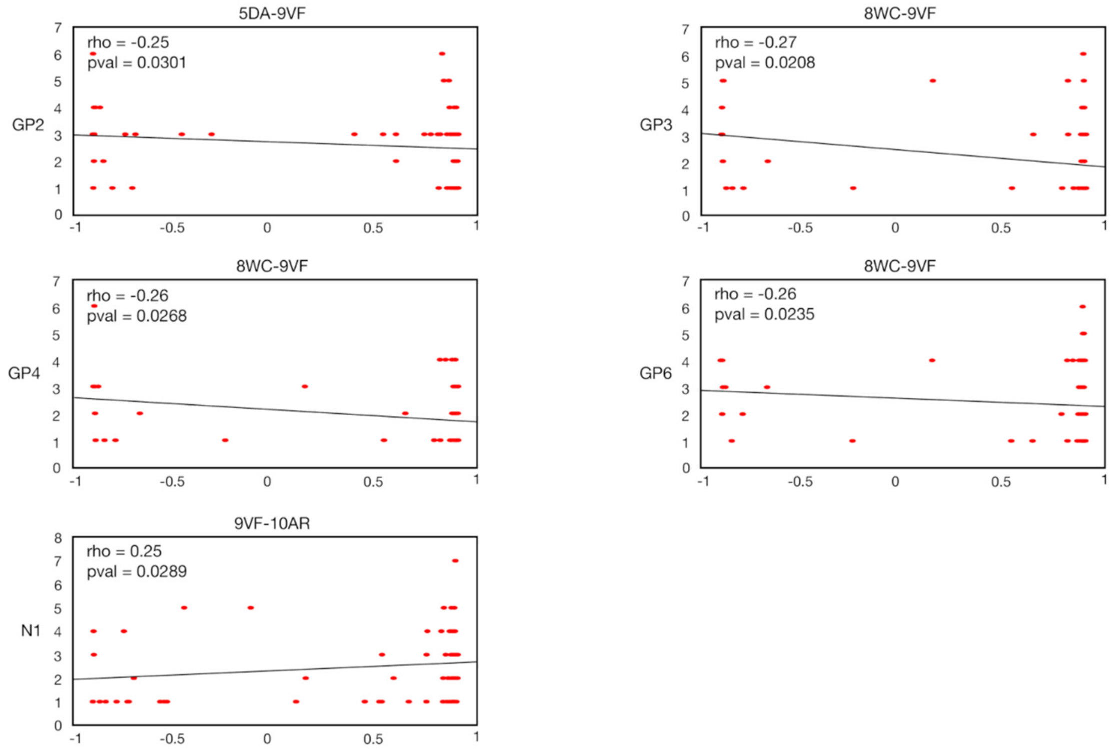

Figure S1. *Correlation plots for significant relationships between fMRI connectivity values and PANSS scores for SZ patients.* The analysis was conducted for the ROIs that presented significant differences between the HC and SZ groups. Spearman's correlation coefficients were calculated ($p < 0.05$, uncorrected). Significant negative relationships between connectivity values of attention- and language-related regions and many items of the General Psychopathology Scale were found, including the GP2 (Anxiety), GP3 (Guilt feelings), GP4 (Tension), and GP6 (Depression) items. A significant positive relationship between 10AR and the N1 (Blunted affect) item of the PANSS was also found.

Correlation between post hoc cerebellar MDTB-based connectivity analysis on patients' subgroup and their PANSS

The correlation analysis with affective and cognitive symptoms (assessed with the PANSS scale) was also separately performed for each subgroup, to identify specific relationships depending on patients' vulnerability to hallucinate. Correlation plots in Fig. S2 depict the significant relationships obtained with $p < 0.05$ (uncorrected).

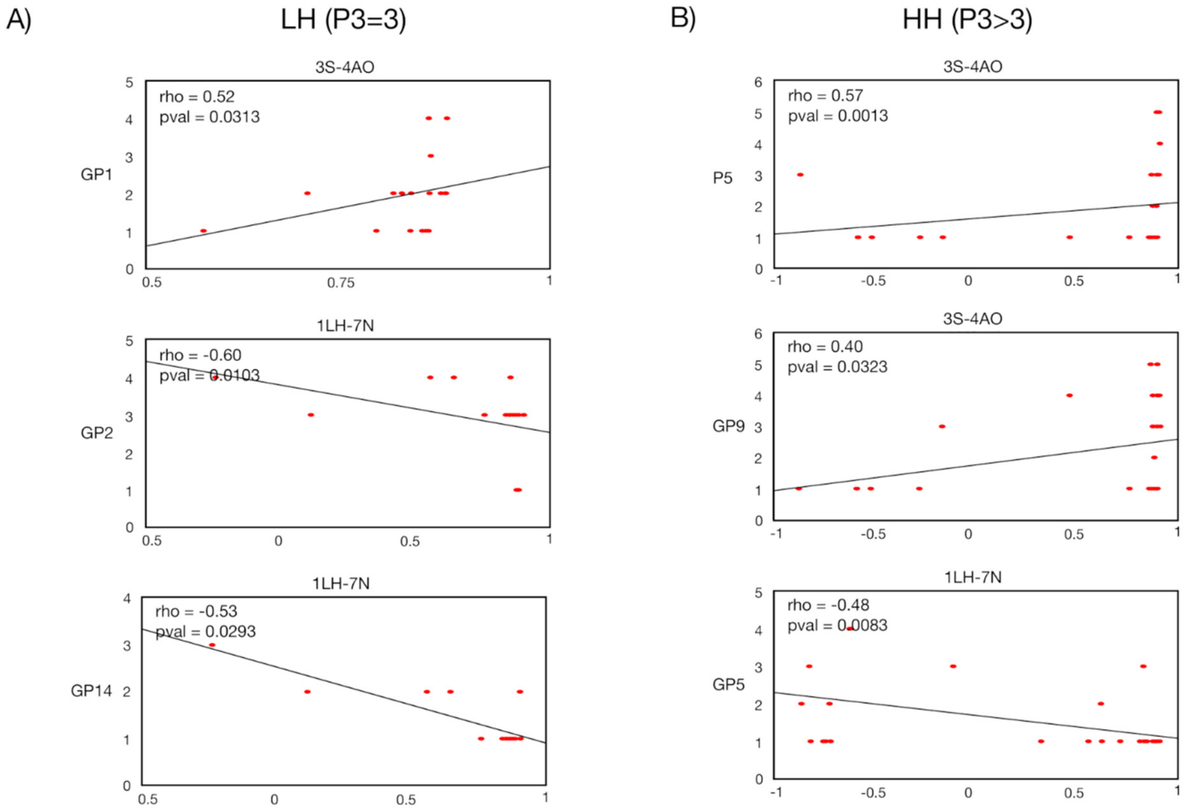

Figure S2. *Correlation plots for significant relationships between fMRI connectivity values and PANSS scores for SZ patients, separately considered according to their subgroups.* The analysis was conducted for the ROIs that presented significant differences between the SZ subgroups, including Low-Hallucinating (LH) and High-Hallucinating (HH) SZ patients. Spearman's correlation coefficients were calculated ($p < 0.05$, uncorrected). Positive associations between 3S-4AO and the GP1 (Somatic concern), GP9 (Unusual thought content), and P5 (Grandiosity) items, and negative associations between 1LH-7N and the GP2 (Anxiety), GP5 (Mannerisms and posturing), and GP14 (Poor impulse control) items, depending on the subgroups, were found.

Functional connectivity differences between HC group and SZ subgroups

We also performed an exploratory comparison between HC group and each SZ subgroup. As can be seen in Fig. S3, the differences between the HC and the SZ groups are partially ascribed to the HH group, as the 5DA-9VF and 8WC-9VF pairs persists in the subgroup comparison. At the same time, a strong difference also persists between the HC and the LH groups for the 8WC-9VF pair. This eventually confirms the possible key role of these cerebellar regions in the emergence of auditory verbal hallucinations in SZ. Also, the difference in connectivity between the 3S-4AO pair for the HC and HH group comparison (which was also reported for the NH vs. HH and LH vs. HH comparison) suggest that an altered connectivity between these regions might underlie hallucinatory phenomena.

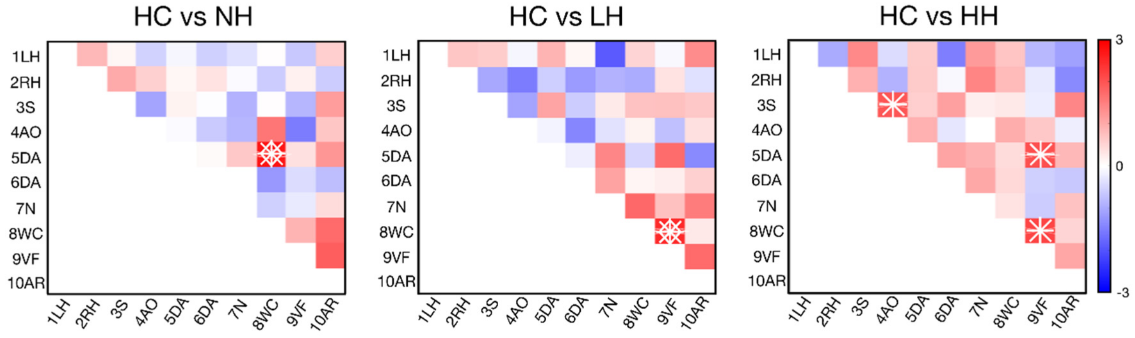

Figure S3. *Functional connectivity differences between all possible pairs of ROIs for the HC group and the SZ subgroups, i.e., Non-Hallucinating (NH), Low-Hallucinating (LH) and High-Hallucinating (HH).* The ten ROIs from the MDTB parcellation were used, and included 1) left-hand presses – 1LH, 2) right-hand presses – 2RH, 3) saccades – 3S, 4) action observation – 4AO, 5) divided attention – 5DA, 6) divided attention – 6DA, 7) narrative – 7N, 8) word comprehension – 8WC, 9) verbal fluency – 9 VF, and 10) autobiographical recall – 10AR. For each ROI pair, significant differences between HC group and SZ subgroups at $p < 0.05$ (uncorrected) are marked with an asterisk, those at $q < 0.05$ (FDR corrected) with a diamond.
